# K_Ca_3.1 channel inhibition leads to an ICAM-1 dependent increase of cell-cell adhesion between A549 lung cancer and HMEC-1 endothelial cells

**DOI:** 10.18632/oncotarget.22735

**Published:** 2017-11-28

**Authors:** Etmar Bulk, Nadzeya Kramko, Ivan Liashkovich, Felix Glaser, Hermann Schillers, Hans-Joachim Schnittler, Hans Oberleithner, Albrecht Schwab

**Affiliations:** ^1^ Institute of Physiology II, University of Muenster, Münster, Germany; ^2^ Institute of Anatomy and Vascular Biology, University of Muenster, Münster, Germany

**Keywords:** NSCLC, extravasation, single cell force spectroscopy, K_Ca_3.1 channels, ICAM-1

## Abstract

Early metastasis leads to poor prognosis of lung cancer patients, whose 5-year survival rate is only 15%. We could recently show that the Ca^2+^ sensitive K^+^ channel K_Ca_3.1 promotes aggressive behavior of non-small cell lung cancer (NSCLC) cells and that it can serve as a prognostic marker in NSCLC. Since NSCLC patients die of metastases, we investigated whether K_Ca_3.1 channels contribute to poor patient prognosis by regulating distinct steps of the metastatic cascade. We investigated the extravasation of NSCLC cells and focused on their adhesion to endothelial cells and on transendothelial migration. We quantified the adhesion forces between NSCLC cells and endothelial cells by applying single cell force spectroscopy, and we monitored transendothelial migration using live-cell imaging. Inhibition of K_Ca_3.1 channels with senicapoc or K_Ca_3.1 silencing increases the adhesion force of A549 lung cancer cells to human microvascular endothelial cells (HMEC-1). Western blotting, immunofluorescence staining and biotinylation assays indicate that the elevated adhesion force is due to increased expression of ICAM-1 in both cell lines when K_Ca_3.1 channels are downregulated. Consistent with this interpretation, an anti-ICAM-1 blocking antibody abolishes the K_Ca_3.1-dependent increase in adhesion. Senicapoc inhibits transendothelial migration of A549 cells by 50%. Selectively silencing K_Ca_3.1 channels in either NSCLC or endothelial cells reveals that transendothelial migration depends predominantly on endothelial K_Ca_3.1 channels.

In conclusion, our findings disclose a novel function of K_Ca_3.1 channels in cancer. K_Ca_3.1 channels regulate ICAM-1 dependent cell-cell adhesion between endothelial and cancer cells that affects the transmigration step of the metastatic cascade.

## INTRODUCTION

Lung cancer is the leading cause of cancer-related death. Of all cancers, the death rates of patients with cancers of the lung and bronchus are the highest in men and women in the United States [1]. Unfortunately, lung cancer is frequently diagnosed at late stages, when distant metastases have already developed. Several steps characterize the metastatic cascade. Cancer cells leave the primary tumor and enter the bloodstream (intravasation). Surviving circulating tumor cells adhere to endothelial cells lining the blood vessels, migrate intravascularly and penetrate the endothelial cell layer (extravasation). Finally, the cells expand to form metastases in different parts of the body [2]. The extravasation of tumor cells bears many similarities with the recruitment of immune cells [3], but the molecular mechanisms underlying tumor cell extravasation are not well understood. Some of the relevant adhesion molecules required for immune cell recruitment, such as LFA-1, have also been found in lung adenocarcinoma cells [4]. Given the importance of tumor metastasis for patient survival and because of limited success of current therapies for non-small cell lung cancer (NSCLC), there is a clear need for the development of new concepts in the understanding of the metastatic cascade.

Alternative approaches have been pursued during the last 15 years after discovering a role for ion channels in tumor cell migration, invasion, adhesion and proliferation. The importance of ion channels in cancer has been firmly established, including NSCLC (see *Phil Trans R Soc B* 2014; 369 (1638) for a series of reviews). Ion channels are commonly expressed aberrantly and/or channel activity is dysregulated in cancer and cancer stroma cells. Thereby, ion channels contribute to the majority of the “hallmarks of cancer” [5]. This also applies to NSCLC. Aberrant expression or dysregulation of K^+^ and other ion channels have been shown and their genes may contain single–nucleotide polymorphisms that predict a poor prognosis [6–8].

There are only few reports indicating that ion channels, in particular Ca^2+^ sensitive K^+^ channels (K_Ca_), are involved in the formation of metastases. The channels K_Ca_2.3 and K_Ca_1.1 promote the development of bone or brain metastases in breast cancer [9, 10]. On a cellular level, the K^+^ channel K_Ca_2.3 (also known as SK3) and the Ca^2+^ channel Orai1 are colocalized in lipid rafts and functionally cooperate in primary tumors to facilitate bone metastasis in breast cancer [9]. Other studies showed that K_Ca_3.1 channels in tumor-associated macrophages promote liver metastases of colorectal cancer by driving cytokine secretion [11]. While these studies provide the proof-of-principle for the involvement of ion channels in the formation of metastases, the underlying mechanisms are still far from being understood. It is, for example, not known which particular steps of the metastatic cascade are driven by these channels. The observation that K_Ca_ channel expression and activity is increased in endothelial cells from clear cell renal and colon carcinoma patients suggests that endothelial ion channels may also be involved in cancer cell dissemination [12, 13]. In this context it is notable that it has long been known that transendothelial migration of neutrophils is accompanied by a rise of the intracellular Ca^2+^ concentration in endothelial cells [14] which has recently been linked to TRPC6 channels [15]. Moreover, adhesion of monocytes to endothelial cells is regulated by K_Ca_1.1 channels [16] and transendothelial migration of lymphocytes into the brain is dependent on endothelial K_2P_2.1 (TREK1) channels [17]. Collectively, these studies lend support to the idea that Ca^2+^ sensitive K^+^ channels are regulators of tumor cell extravasation.

Here, we show that K_Ca_3.1 channels regulate the extravasation of A549 NSCLC cells through an endothelial cell layer by regulating ICAM-1 expression. Interestingly, K_Ca_3.1 channels in endothelial cells appear to be more important for this process than those in NSCLC cells.

## RESULTS

### Inhibition of K_Ca_3.1 channels increases the adhesion force between A549 NSCLC cells and human microvascular endothelial (HMEC-1) cells

**Figure 1:**
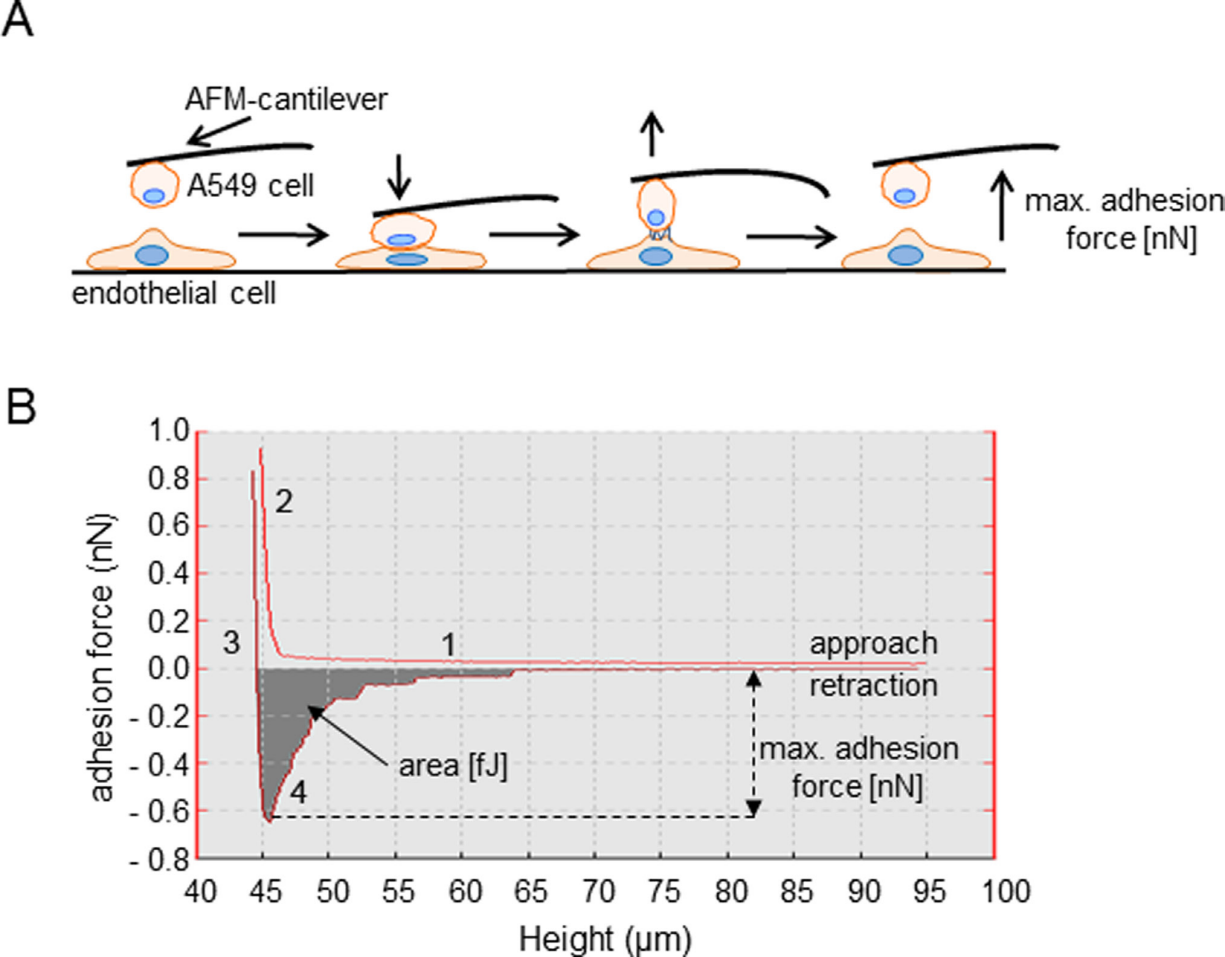
Illustration of adhesion force measurements using single cell force spectroscopy (SCFS) (**A**) Single A549 cells are picked with a WGA-coated AFM-cantilever and brought into contact with HMEC-1 endothelial cells. A constant force (1 nN) is applied to maintain the contact between both cells for a period of 2 s. The force needed to separate the two cells (adhesion force [nN]) is measured during retraction of the cantilever. (**B**) Representative example of a force-distance curve. The characteristic phases of the experiment are indicated by numbers. 1. Approach of the A549 cell to an endothelial cell. No cell-cell contact has been formed. 2. The NSCLC cell is “pressed” onto the endothelial cell, positive force is applied. 3. Retraction of the cantilever so that the A549 cell is lifted off of the endothelial cell. 4. Detachment of the A549 cell from the endothelial cell. The turning point of the curve represents the maximal adhesion force [nN], and the shaded area under the curve is a measure of the detachment work [fJ].

**Figure 2:**
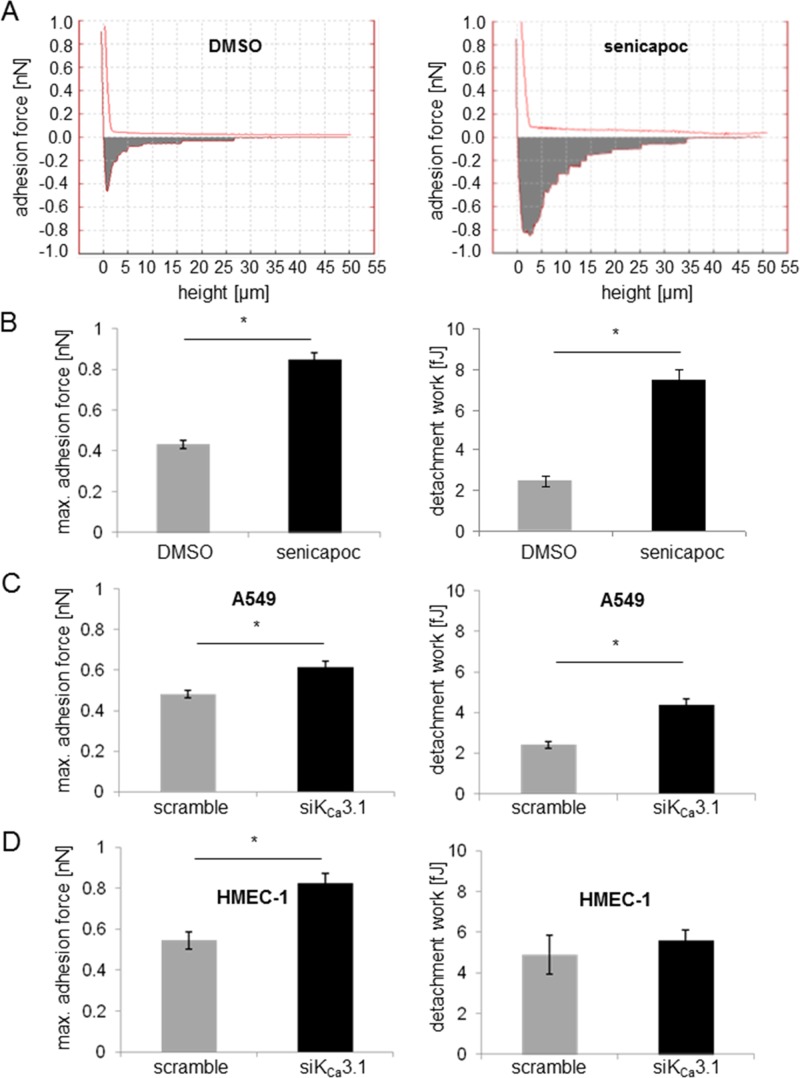
Inhibition of K_Ca_3.1 channels increases the adhesion force between A549 NSCLC and HMEC-1 endothelial cells The adhesion force is measured using single cell force spectroscopy. (**A**) Typical force-distance curves recorded under control conditions (vehicle DMSO; left) and in the presence of the K_Ca_3.1 channel blocker senicapoc (right). Summary of the adhesion force measurements between A549 and HMEC-1 cells. Data are obtained from *N* = 9 independent experiments. Each experiment has been performed such that any given A549 cell, attached to the AFM-cantilever, has come into contact with 20 different endothelial cells. ^*^denotes *p* < 0.05. (**B**) Adhesion force measurements were performed under control conditions or in the presence of senicapoc. (**C**) A549 cells were transfected with siRNA against K_Ca_3.1 channels and used for experiments 48 h after transfection. (**D**) HMEC-1 endothelial cells were transfected with siRNA against K_Ca_3.1. 48 hours after transfection, the cells were incubated for 24 hours with 10 ng/ml TNF-α.

Extravasation is a crucial step of the metastatic cascade of NSCLC cells. It is preceded by adhesion of NSCLC cells to the vascular endothelium. We employed single cell force spectroscopy to investigate how adhesion of NSCLC cells to endothelial cells is regulated by K_Ca_3.1 channels. We blocked K_Ca_3.1 channels using either the inhibitor senicapoc or silencing with siRNA. Figure 1 depicts a sketch of the experimental procedures. An A549 cell attached to the cantilever of the AFM (atomic force microscope) is brought into contact with an HMEC-1 cell for 2 s. The force needed to separate the newly formed cell-cell contacts between A549 and HMEC-1 cells is measured while lifting the AFM cantilever (Figure 1). The first measurements were performed in the presence of senicapoc or its solvent DMSO. Under control conditions (DMSO) the maximal adhesion force and the detachment work amount to 0.43 ± 0.02 nN and 2.4 ± 0.26 fJ, respectively (*N* = 9 experiments with *n* = 20 HMEC-1 cells). These values strongly increase to 0.85 ± 0.03 nN and 7.5 ± 0.51 fJ in the presence of the K_Ca_3.1 blocker senicapoc (Figure 2A and 2B; (*N* = 9 experiments with *n* = 20 HMEC-1 cells).

We next tested whether the increase in adhesion force upon K_Ca_3.1 inhibition is due to K_Ca_3.1 channel inhibition in A549 cells or in endothelial (HMEC-1) cells because it is well established that K_Ca_3.1 channels are expressed in both cell lines [6, 18]. Therefore, we performed single cell force spectroscopy with A549 cells that were transiently transfected with siRNA against K_Ca_3.1. The adhesion force between A549 and HMEC-1 cells increases, but not as markedly as in the presence of senicapoc (Figure 2C). Adhesion force is 29% higher in siK_Ca_3.1 transfected A549 cells (0.62 ± 0.03 nN) than in control transfected (scramble) cells (0.48 ± 0.02 nN; *N* = 9 with *n* = 20 HMEC-1 cells). Similarly, the detachment work increases, but not as strongly as in the presence of senicapoc: 4.4 ± 0.31 fJ for siK_Ca_3.1 transfected cells versus 2.4 ± 0.17 fJ for scramble transfected cells.

In the next set of experiments we used HMEC-1 cells that were transfected with siRNA against K_Ca_3.1. The K_Ca_3.1 knockdown cells were probed with untreated A549 cells. Again, maximal adhesion force increases. The respective values are 0.82 ± 0.05 nN versus 0.54 ± 0.04 nN for siK_Ca_3.1-HMEC-1 and control (scrambled) cells, respectively (Figure 2D). The detachment work does not change (5.6 ± 0.51 fJ versus 4.9 ± 0.96 fJ for siK_Ca_3.1-HMEC-1 and control cells, respectively; Figure 2D; *N* = 9 with *n* = 20 HMEC-1 cells).

### Downregulation of K_Ca_3.1 increases ICAM-1 expression

**Figure 3:**
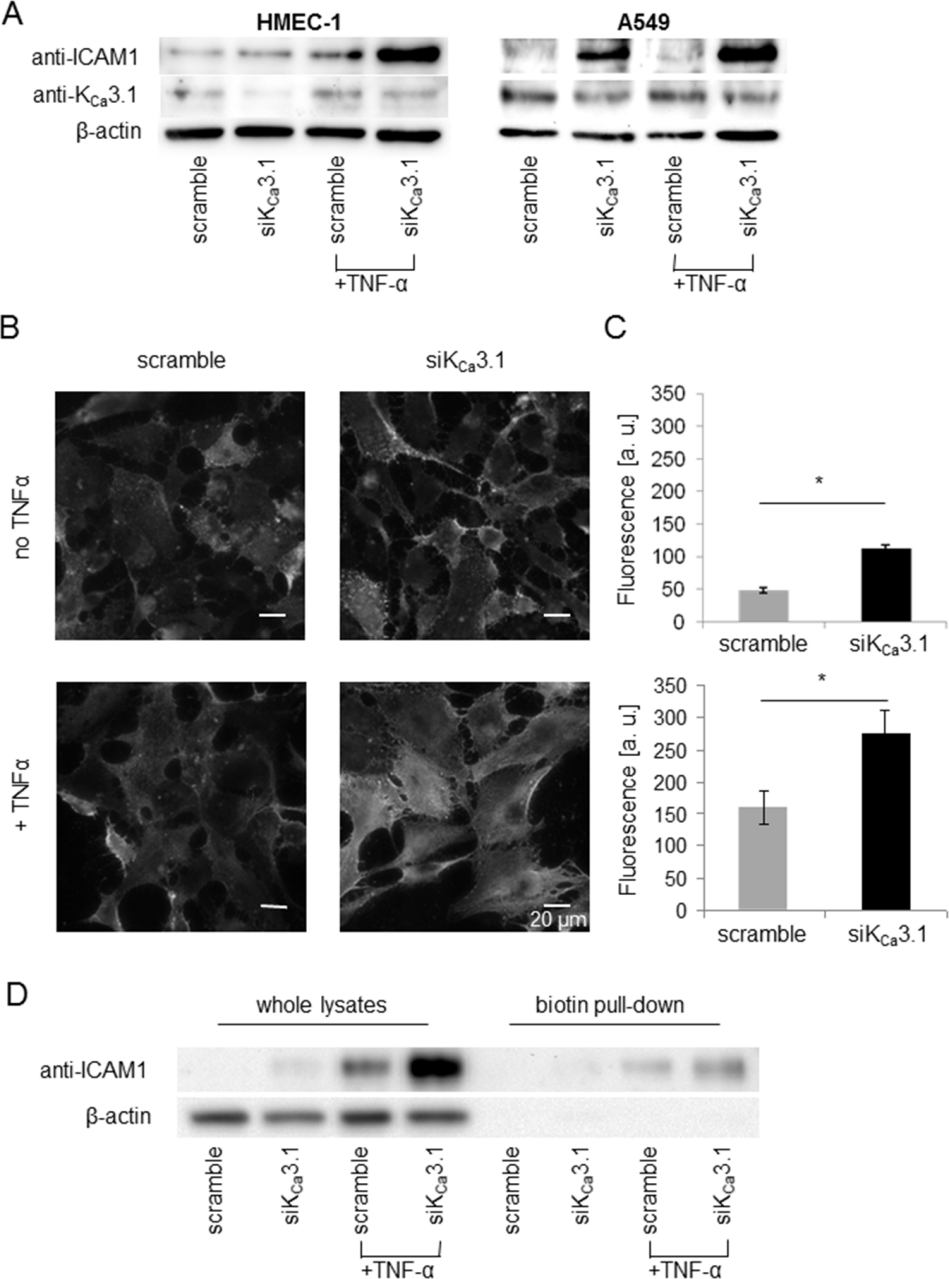
Downregulation of K_Ca_3.1 increases ICAM-1 expression in HMEC-1 endothelial and A549 NSCLC cells (**A**) Western blots of whole cell lysates from HMEC-1 and A549 cells transfected with control siRNA (scramble) or siRNA targeting K_Ca_3.1 channels (siK_Ca_3.1). The blot is representative for three replicates. (**B**) ICAM-1 staining of non-permeablized HMEC-1 endothelial cells with an anti-ICAM-1 antibody recognizing an extracellular epitope. (**C**) Quantification of ICAM-1 fluorescence intensity [arbitrary units a.u.]; *n* = 30 cells from *N* = 3 experiments. ^*^denotes *p* < 0.05. (**D**) ICAM-1 expression in membrane fractions of HMEC-1 endothelial cells obtained from biotinylation assays. We blotted the fraction of biotinylated membrane proteins pulled down with streptavidin beads (biotin) and proteins from the remaining cell lysates (lysate) of HMEC-1 cells, treated with and without TNF-α. Actin blots validate the surface biotinylation because no actin is detected in the fraction of biotinylated membrane proteins. The blots are representative for four experiments.

After showing that K_Ca_3.1 channels regulate cell-cell adhesion between NSCLC and endothelial cells, we investigated potential underlying mechanisms. By analogy with immune cell recruitment that involves intercellular adhesion molecule 1 (ICAM-1) [19], we tested whether ICAM-1 has any impact on the adhesion between A549 and HMEC-1 cells and its regulation by K_Ca_3.1 channels. Using siRNA and protein expression analysis, we investigated whether ICAM-1 expression is modulated by K_Ca_3.1 channels (Figure 3). HMEC-1 and A549 cells were transiently transfected with siRNA against K_Ca_3.1. 24 hours, before lysis, cells were pretreated with (+TNF-α) or without TNF-α (10 ng/ml), known to induce surface expression of ICAM-1. Western blots reveal a strong increase of ICAM-1 expression when K_Ca_3.1 channels are silenced in HMEC-1 cells (Figure 3A, left figure). Interestingly, we observe a similar effect in A549 cells. K_Ca_3.1 downregulation in these cells also leads to an increased expression of ICAM-1 (Figure 3A, right figure). No ICAM-1 expression is detectable in A549 cells transfected with the scrambled control siRNA.

To complement the above results obtained with whole-cell lysates, we also determined ICAM-1 expression in the plasma membrane of HMEC-1 cells. We used immunofluorescence employing an ICAM-1 antibody that binds to an extracellular epitope (Figure 3B and 3C). The fluorescence intensity of siK_Ca_3.1-transfected HMEC-1 cells immunostained for ICAM-1 is significantly higher than in control cells. Fluorescence intensity (given in arbitrary units, a. u.; Figure 3C) rises from 48.0 ± 3.6 a. u. in scramble-transfected cells (*N* = 3 with *n* = 30 cells) to 112.0 ± 5.8 a. u. in siK_Ca_3.1-transfected cells (*N* = 3 with *n* = 30 cells) when HMEC-1 cells are not pretreated with TNF-α. Following TNF-α pretreatment, the respective values are 159.0 ± 26.4 a. u. and 274.0 ± 37.2 a. u. for scramble-transfected and siK_Ca_3.1-transfected cells (*N* = 3 with *n* = 30 cells).

To verify that ICAM-1 is present on the cell surface of HMEC-1 endothelial cells, we performed surface biotinylation assays and analyzed ICAM-1 expression in the plasma membrane fraction using Western blotting. Figure 3D shows that ICAM-1 is expressed in the plasma membrane, especially in samples from siK_Ca_3.1-transfected cells.

Collectively, Western blotting, immunofluorescence staining, and surface biotinylation reveal a strong upregulation of ICAM-1 expression in the plasma membrane of HMEC-1 cells when K_Ca_3.1 channels are downregulated. This is consistent with the idea that ICAM-1 is a mediator of the K_Ca_3.1-dependent increase in cell-cell adhesion forces between A549 and HMEC-1 cells.

### Inhibiton of ICAM-1 prevents K_Ca_3.1 channel-dependent strengthening of cell-cell adhesions

**Figure 4:**
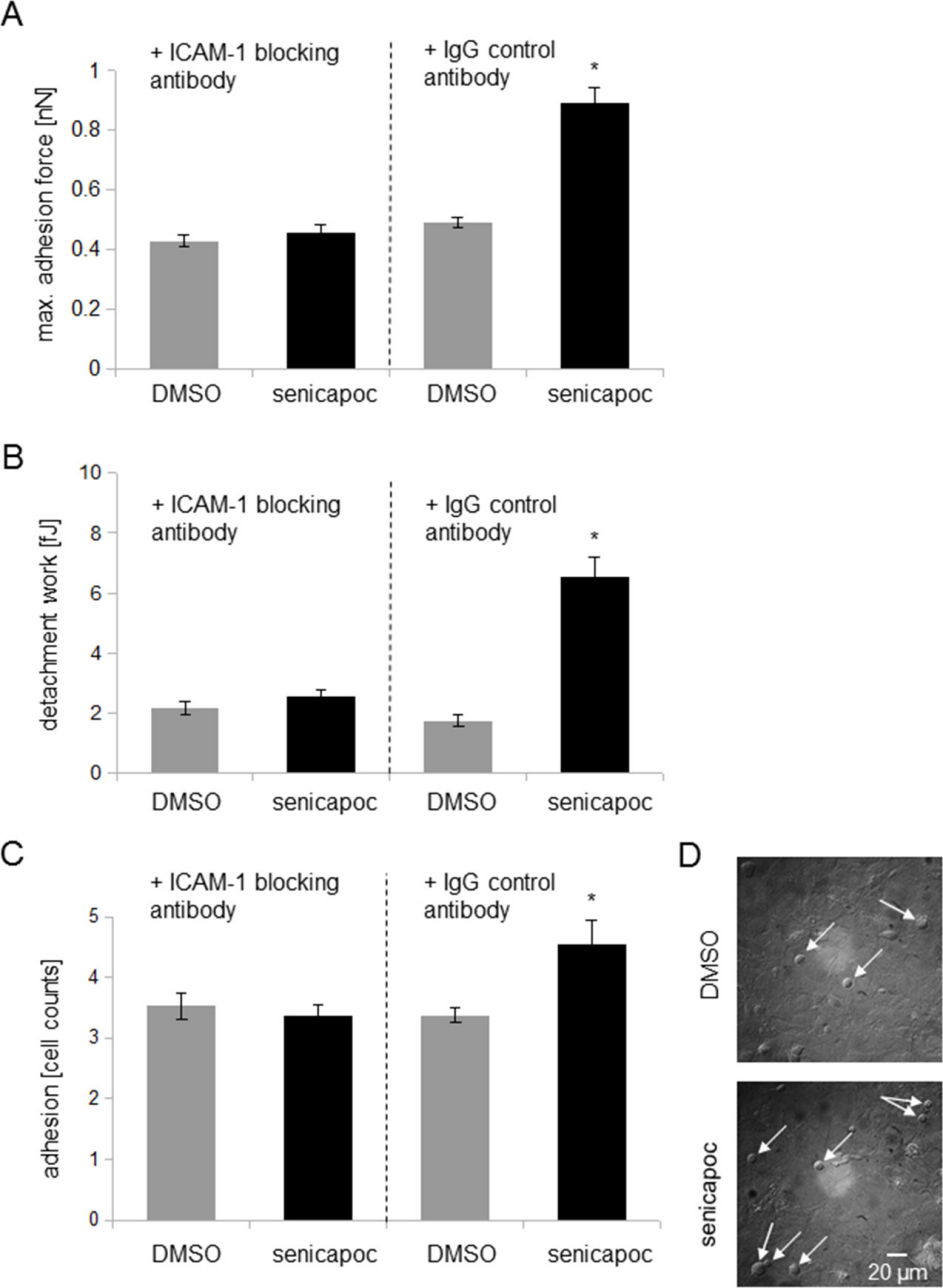
Inhibiton of ICAM-1 prevents K_Ca_3.1 channel-dependent strengthening of adhesion (**A**) and (**B**) Adhesion measurements using single cell force spectroscopy. The experimental procedures are shown in Figure 1. Prior to the measurements, A549 cells were incubated for 30 min with 10 μg/ml ICAM-1 blocking antibody or an IgG control antibody. (**A**) Measurements of the maximum adhesion force [nN]. (**B**) Detachment work [fJ] that is needed to seperate A549 cells from the endothelial cells. *N* = 7 independent experiments, in which A549 cells were brought into contact with 20 different HMEC-1 endothelial cells each. ^*^p < 0.05. (**C** and **D**) Cell-cell adhesion of A549 to HMEC-1 endothelial cells. (**C**) A549 cells pre-incubated either with 10 μg/ml ICAM-1 blocking antibody or with an IgG control antibody (8 μg/ml) were seeded for 2.5 hours on a confluent endothelial layer. After washing and fixation, adherent A549 cells were quantified via microscopy. *N* = 6 independent experiments, and 10 different image sections per experiment were analysed. ^*^denotes *p* < 0.05. (**D**) Representative images of adherent A549 cells on a confluent HMEC-1 endothelial layer (white arrows indicate A549 cells).

If ICAM-1 mediates the K_Ca_3.1 channel-dependent increase in adhesion between A549 and HMEC-1 cells, this effect will be abolished by ICAM-1 inhibition. To test this idea we performed additional single cell force spectroscopy experiments. A549 cells were preincubated with a monoclonal ICAM-1 blocking antibody or an IgG control antibody. Thereafter, the cells were attached to a cantilever, and the effect of senicapoc was evaluated. In the presence of the blocking ICAM-1 antibody, senicapoc fails to induce an increase in adhesion forces between A549 and HMEC-1 cells (Figure 4A and 4B). Adhesion forces amount to 0.43 ± 0.02 nN (control, DMSO; *N* = 7 with *n* = 20 HMEC-1 cells) and 0.46 ± 0.03 nN (senicapoc; *N* = 7 with *n* = 20 HMEC-1 cells). Similarly, detachment work is unchanged (DMSO = 2.15 ± 0.2 fJ; senicapoc = 2.53 ± 0.22 fJ). In contrast, adhesion force measurements using the IgG control antibody show a similar effect as obtained previously. The adhesion force under control conditions using DMSO amounts to 0.49 ± 0.02 nN, and in the presence of senicapoc, the adhesion force increases to 0.89 ± 0.05 nN (*N* = 7 with *n* = 20 HMEC-1 cells). The detachment work increases from 1.73 ± 0.19 fJ under control conditions (DMSO) to 6.53 ± 0.66 fJ, following treatment with senicapoc.

To strengthen these findings, we used an additonal approach by quantifiying the adherence of A549 cells to a confluent endothelial cell layer. Similar to the adhesion measurements, A549 cells were incubated with a blocking ICAM-1 antibody or an IgG control antibody. Then, A549 cells were seeded onto a confluent endothelial cell layer and after 2.5 hours incubation time the supernatant was removed. Thereafter, adherent A549 cells were analysed with a microscope. In the presence of an IgG control antibody, A549 cell adhesion is increased with senicapoc treatment (4.55 ± 0.4 A549 cells, Figure 4C and 4D). In contrast, under control conditions with DMSO 3.83 ± 0.13 A549 cells adhered to the endothelial cell layer. In the presence of ICAM-1 blocking antibody, no difference in A549 cell adhesion is oberservable, neither in the presence of senicapoc (3.83 ± 0.167 A549 cells) nor its solvent DMSO (3.53 ± 0.21 A549 cells).

These findings further support the idea that ICAM-1 is one of the mediators of K_Ca_3.1-dependent adhesion between A549 lung cancer cells and HMEC-1 endothelial cells.

### Transendothelial migration of A549 lung cancer cells is inhibited by the K_Ca_3.1 channel blocker senicapoc

**Figure 5:**
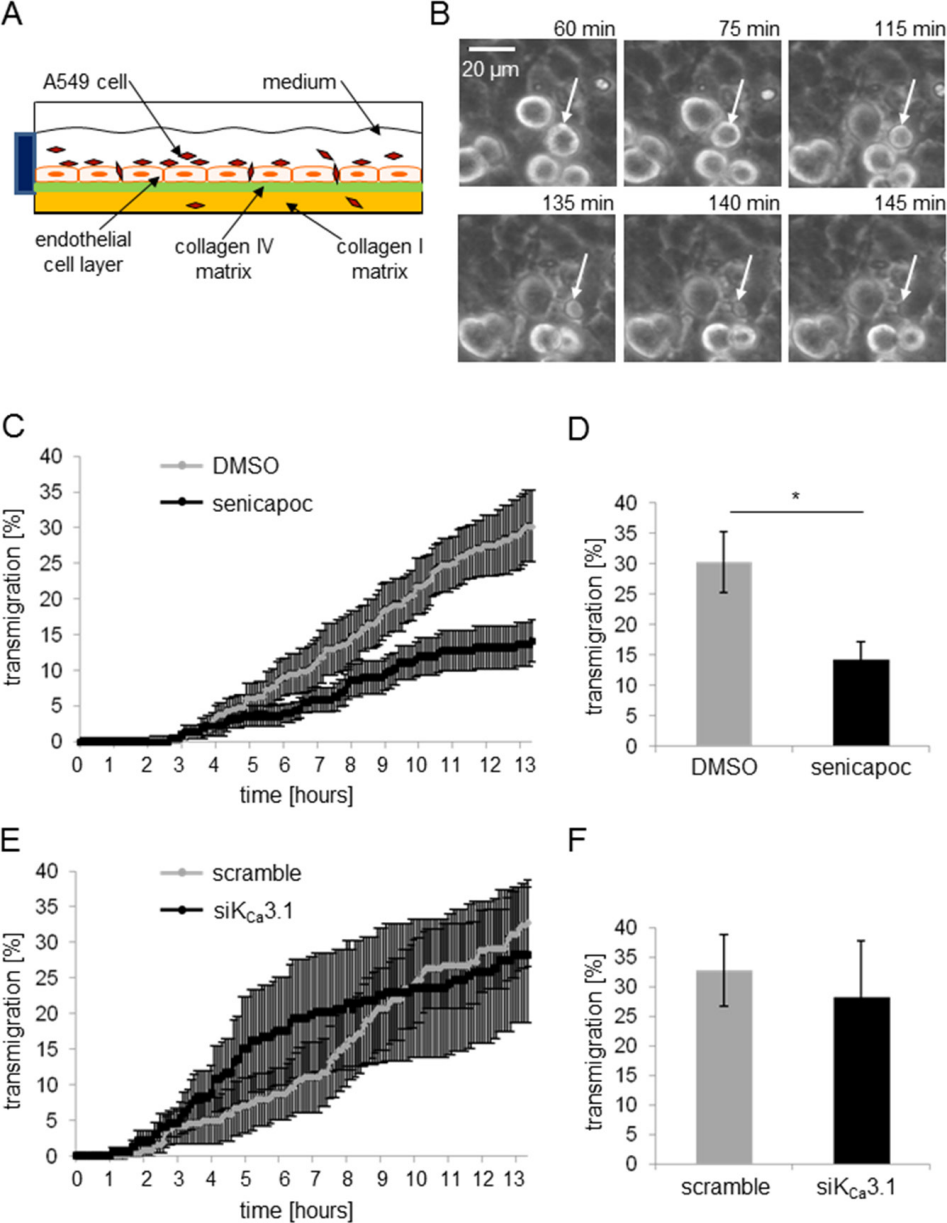
The K_Ca_3.1 channel blocker senicapoc inhibits invasion of A549 cells through an endothelial cell layer (**A**) Invasion assay. HMEC-1 endothelial cells are seeded on a “sandwiched” collagen matrix consisting of a thick cushion of collagen I superimposed by a thin reconstituted “basememt membrane” containing collagen IV. After endothelial cells have grown to confluency, they are incubated with TNF-α (10 ng/ml) for 24 hours. The following day, A549 cells are added and transendothelial migration is monitored for 13.5 h using live-cell imaging. (**B**) Series of images of an A549 cell transmigrating through the endothelial cell layer. White arrows indicate the transmigrating A549 cell. (**C**–**F**) Analysis of the transmigration experiments. The time course of the experiments is plotted in (**C**) and (**E**), the summary of the experiments in (**D**) and (**F**). *N* = 6 experiments each. (**C**) and (**D**) Invasion of A549 cells in the presence of DMSO (control conditions) or 30 μmol/l senicapoc. ^*^denotes *p* < 0.05. (**E**) and (**F**) Invasion of A549 cells transfected with siRNA against K_Ca_3.1 channels.

In the following, we employed an *in vitro* “extravasation” assay (Figure 5A) to test whether the altered adhesion of NSCLC cells in response to K_Ca_3.1 channel inhibition also affects transendothelial migration. Using live-cell imaging, we were able to follow A549 lung cancer cells on their way through an endothelial cell layer formed by HMEC-1 cells (Figure 5B). In the first set of experiments we monitored transendothelial migration of A549 cells in the presence of senicapoc or its solvent DMSO. The time course of these experiments is depicted in Figure 5C. The percentage of A549 cells that transmigrate through the endothelial layer is plotted as a function of time. Transmigration starts after a lag period of ~3 hours. Under control conditions A549 cells continuously transmigrate so that the relative number of these cells steadily increases to 30 ± 5% at *t* = 13.5 h (*N* = 6). In the presence of senicapoc, transmigration of A549 cells is much slower. Figure 5D provides the statistical evaluation of the end point of the experiments at *t* = 13.5 h. K_Ca_3.1 channel inhibition reduces the transendothelial migration by half. In the presence of senicapoc the number of transmigrated A549 cells is reduced to 14 ± 3%.

Next, we repeated transmigration experiments with siK_Ca_3.1-transfected A549 cells (Figure 5E and 5F). Inspection of Figure 5E reveals a different time course as compared to the experiments performed with senicapoc. Transmigration of siK_Ca_3.1-transfected A549 cells starts approximately 1 hour earlier and accelerates during the first 8 h. At the end of the experiment, however, there is no difference between A549 cells transfected with siK_Ca_3.1-RNA or scrambled siRNA. 28 ± 6% of siK_Ca_3.1-RNA transfected and 33.0 ± 9.5% of scrambled RNA transfected cells, respectively, transmigrate through the endothelial cell layer (Figure 5F). This led us to conclude that K_Ca_3.1 channels in endothelial cells, rather than in NSCLC cells, are more important for transendothelial migration. We therefore needed to test whether K_Ca_3.1 channel inhibition affects endothelial barrier integrity.

### Blocking of K_Ca_3.1 channels shows no impact on the permeability of the endothelium

**Figure 6:**
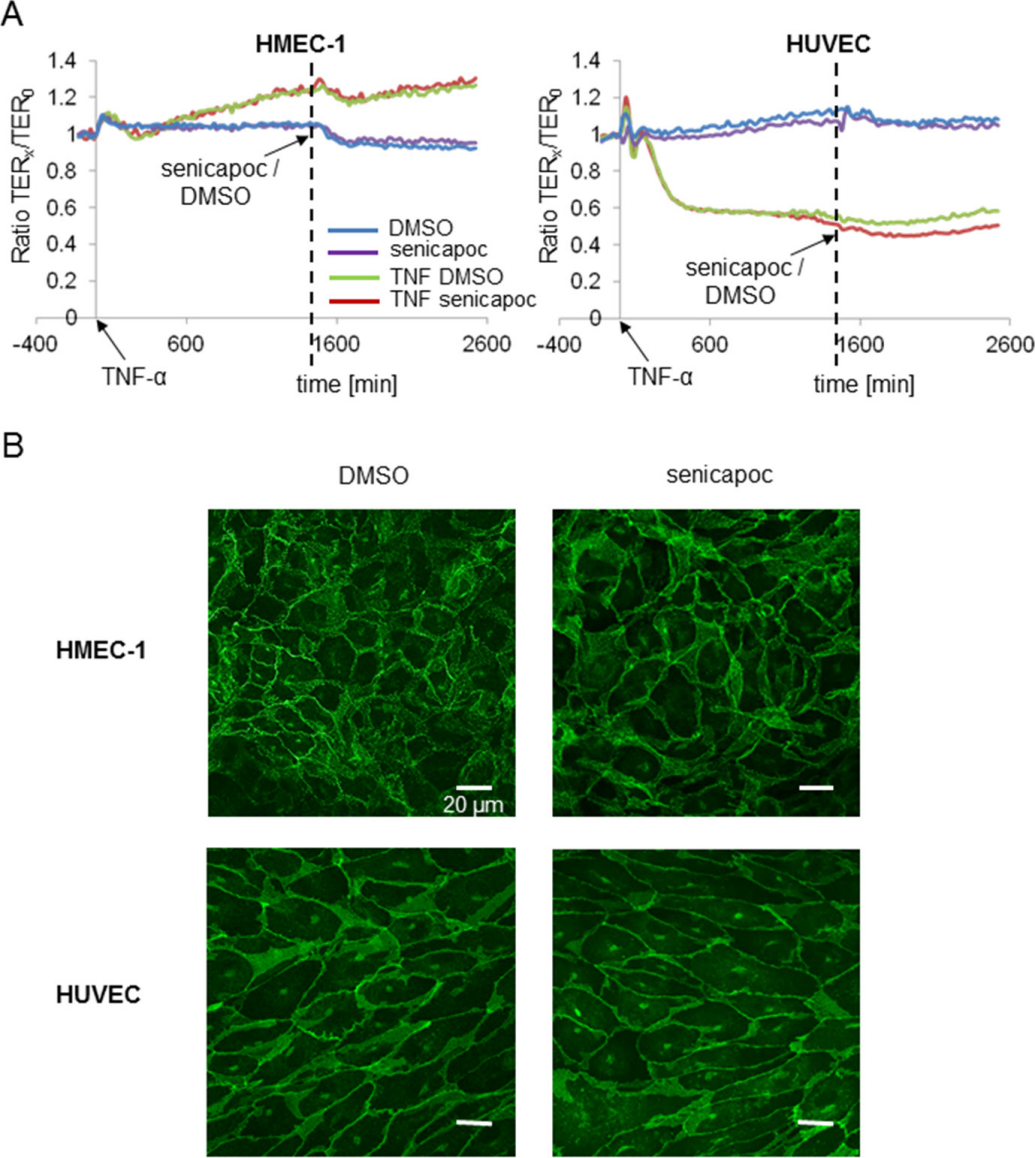
Transendothelial resistance measurements (TER) with HMEC-1 and HUVEC cells showing no impact of senicapoc on endothelial barrier function HMEC-1 and HUVEC cells are seeded in an impedance chamber equipped with a glass surface coated with 0.5% crosslinked gelatin. When confluent, cells are treated with or without 100 ng/ml TNF-α. After 24 hours of incubation with/without TNF-α, 30 μmol/l of senicapoc or DMSO are added. (**A**) Time courses of TER-measurements of HMEC-1 and HUVEC cells. The time point “0” reproduces the addition of TNF-α. The vertical broken line indicates the addition of senicapoc or DMSO. (**B**) Representative images of immunofluorescence stainings of HMEC-1 and HUVEC cells, treated with senicapoc or DMSO. Cells were stained for VE-cadherin.

We measured the transendothelial resistance (TER) of confluent HMEC-1 cells. For comparison, we also measured the TER of human umbilical cord vein endothelial cells (HUVEC). Resistance values were taken every 20 minutes, beginning directly after the addition of 100 ng/ml TNF-α. Approximately 24 hours later, senicapoc or its solvent DMSO were added. The results are summarized in Figure 6A. Resistance values are normalized to the TER at *t* = 0 (TER_0_). TNF-α affects both cell lines in opposite ways. TNF-α leads to an increase of the resistance of HMEC-1 cells by ~25%, while the TER of HUVEC cells decreases by ~50%. However, it is important to note that neither the vehicle DMSO nor senicapoc cause any significant change of the transendothelial resistance in either cell line. Impedance analysis was complemented by immunofluorescence stainings of VE-cadherin. Senicapoc shows no impact on the staining patterns in either cell line (Figure 6B). These findings indicate that K_Ca_3.1 channel-dependent inhibition of transendothelial migration cannot be accounted for by altered barrier function of the endothelial layer.

## DISCUSSION

Extravasation is a crucial step of the metastatic cascade. One way for tumor cells to extravasate and form hematogenous metastases is that they, like immune cells, adhere to endothelial cells and then transmigrate through the endothelial barrier. Adhesion to and transmigration through the endothelial barrier have been studied in great detail in immune cells, but to a lesser extent in tumor cells. So far, the role of ion channels in tumor or immune cell transmigration has hardly been investigated in detail. Neutrophil recruitment is regulated by TRPC6 channels in endothelial cells [15]. Transendothelial migration of lymphocytes into the brain is regulated by endothelial K_2P_2.1 channels; it is enhanced in K_2P_2.1^−/−^ mice [17]. To the best of our knowledge, there are no studies directly showing an involvement of ion channels in the process of tumor cell extravasation. Thus, our study helps to fill this gap of knowledge.

The key results of our study are the following. (i) K_Ca_3.1 channel inhibition or silencing in NSCLC and endothelial cells increases the adhesion force between the two cell types. (ii) K_Ca_3.1 silencing leads to an upregulation of ICAM-1 in NSCLC and endothelial cells. (iii) K_Ca_3.1-dependent increase of adhesion forces between A549 and HMEC-1 cells is prevented by blocking ICAM-1. (iv) Blocking K_Ca_3.1 channels inhibits transmigration of NSCLC cells through an endothelial cell layer. Since transmigration is only marginally affected by silencing K_Ca_3.1 channels in NSCLC cells, endothelial K_Ca_3.1 channels appear to be more important for the transmigration step. This view is supported by the adhesion measurements which show a stronger increase in adhesion force upon silencing of K_Ca_3.1 channels in HMEC-1 cells as compared to that in A549 cells.

At first sight, these findings appear to be contradictory and to argue against K_Ca_3.1 channels being a potential therapeutic target in NSCLC metastasis. K_Ca_3.1 channels maintain endothelial and NSCLC cells in a state of low to intermediary adhesiveness which is permissive for transendothelial migration of NSCLC cells. This is in line with the observation that an aggressive metastasising strain of A549 cells is characterized by low adhesiveness [20]. We reported earlier that the metastasising A549 cell strain has elevated K_Ca_3.1 expression [6].

K_Ca_3.1 inhibition renders the endothelium more adhesive for NSCLC cells, but impairs the transmigration of NSCLC cells. Thus, K_Ca_3.1 channels have distinct effects on these two steps of the extravasation cascade. By analogy with immune cells, it has to be kept in mind that cells once adhering to the endothelium must migrate to reach the site of emigration. Selectively interfering with either adhesion or migration will lead to a reduced number of transmigrated cells. This has been shown in the case of neutrophil transmigration by either deleting LFA-1 or Mac-1, which are involved in neutrophil adhesion to and migration on endothelial cells, respectively [21, 22]. Along these lines it is feasible that K_Ca_3.1 channel inhibition is able to attenuate metastasis because the strongly enhanced adhesion impairs the ability of NSCLC cells to reach their site of transmigration.

This view is supported by previous findings. Migration has a bell-shaped dependence on the adhesion force. Migration is fast at intermediary and slow at increased or reduced adhesion strength [23]. Finally, K_Ca_3.1 channel inhibition may be therapeutically beneficial by increasing homotypic adhesion between NSCLC cells and thereby preventing the dissemination of NSCLC cells from the primary tumor. Such a behavior has been observed in melanoma cells. Metastatic spread from the primary tumor was low when cell-cell adhesion was high [24]. In this context it is interesting to note that the therapeutic reduction of cell-cell adhesion by inhibition of VLA-4 (α4β1 integrin; ligand of endothelial VCAM-1) in multiple sclerosis patients may elevate the risk to develop melanoma (e.g. [25]). Similarly, down-regulation of ICAM-1 enhanced liver metastasis of pancreatic cancer [26].

An alternative mechanism for impaired transmigration in response to K_Ca_3.1 channel inhibition/knockdown may relate to the endothelial barrier integrity. Leukocyte transmigration is accompanied by an elevation of the intracellular Ca^2+^ concentration in endothelial cells [14]. Increasing the intracellular Ca^2+^ concentration, e.g. by activating TRPV4 channels, has been shown to cause disintegration of the endothelial barrier function, which could be prevented by K_Ca_3.1 channel inhibition [27].

So far, we do not know the mechanisms by which K_Ca_3.1 channel silencing causes an upregulation of ICAM-1. Notably, endothelial K_Ca_3.1 channels have the same function as K_2P_2.1 channels in this respect. Knockout of K_2P_2.1 channels in endothelial cells of the blood brain barrier is also accompanied by an upregulation of ICAM-1 [17]. Similarly, both K_2P_2.1 channel knockout and K_Ca_3.1 channel inhibition do not affect the endothelial barrier function as assessed by impedance analysis. The most evident common denominator of K_Ca_3.1 and K_2P_2.1 channel function is their impact on the cell membrane potential. Channel inhibition causes membrane depolarization which decreases the driving force for Ca^2+^ entry and will lead to a decrease in intracellular Ca^2+^ concentration [27]. This, however, cannot account for increased ICAM-1 expression. ICAM-1 expression is regulated among other factors by NF-κB, which is activated by a rise of the intracellular Ca^2+^ concentration [28]. In addition, ICAM-1 expression is also regulated by ROS production [29], which in turn is modulated by mitochondrial K^+^ channels [30]. While the expression of K_Ca_3.1 channels in the inner membrane of mitochondria has been shown [31], it is still elusive whether mitochondrial K_Ca_3.1 inhibition in NSCLC cells enhances ROS production and thereby increases ICAM-1 expression.

Taken together, our results show that K_Ca_3.1 channels not only regulate the behavioral traits of tumor cells, such as their ability to migrate and proliferate, but they also contribute to more complex steps of the metastatic cascade, such as tumor cell extravasation. One of the key findings of our study is the importance of K_Ca_3.1 channels in endothelial cells for NSCLC cell extravasation. In our view, these findings provide evidence for evaluating K_Ca_3.1 channels as therapeutic target in NSCLC. The K_Ca_3.1 blocker senicapoc, used in our study, is well tolerated in patients [32].

## MATERIALS AND METHODS

### Cell culture

All experiments were performed with highly metastatic A549 lung adenocarcinoma cells which were derived from the original A549 cell line (ATCC^®^CCL-185^™^). The isolation of this cell line has been described elsewhere [33]. A549 cells were cultured at 37°C, 5% CO_2_ in DMEM medium (Dulbecco's Modified Eagle's medium, Sigma-Aldrich) supplemented with 10% Fetal Bovine Serum (FBS Superior, Biochrom GmbH, Germany). The human microvascular endothelial cell line HMEC-1 was cultured on 0.5% gelatine-coated substrates and maintained in MCDB medium (ThermoFisher Scientific), supplemented with 10% Fetal Bovine Serum Gold (FBS, GE Healthcare Europe GmbH, Germany), 1% Glutamax (ThermoFisher Scientific), 1% Penicillin/ Streptomycin (Merck Millipore), 10 ng/ml epidermal growth factor (EGF, Sigma-Aldrich), and 1 μg/ml hydrocortison (Sigma-Aldrich).

Human umbilical cord vein endothelial cells (HUVEC) were isolated from anonymized donors as described [34] according to the principles outlined in the Declaration of Helsinki; this was approved by the ethics boards of the University of Münster (2009-537-f-S, respectively). Cells of passage 1 were used for experiments and cultured on cross-linked gelatine as described elsewhere [35].

### siRNA transfection

Transfection of A549 or HMEC-1 cells was carried out in Opti-MEM I Reduced Serum Media (ThermoFisher Scientific). Specific siRNA against K_Ca_3.1 or a universal scrambled control (Origene, Rockville, USA, final concentration 2 nM, Supplemental Material and Methods) were transfected transiently, using the Lipofectamine RNAiMAX Transfection Reagent (ThermoFisher Scientific) according to the manufacturer´s protocol. Experiments were performed 48 h after transfection.

### Western blot analysis

Cells were lysed in RIPA buffer buffer (50 mmol/l Tris, 150 mmol/l NaCl, 0.1% SDS, 0.5% sodium deoxycholate, 1% NP-40 and protease inhibitors from Roche, Germany). PVDF-membranes were blocked in PBS containing 5% skim milk and 0.05% Tween (Sigma-Aldrich). We used the following antibodies: rabbit anti-K_Ca_3.1 (1:1000, # AV35098, Sigma-Aldrich, USA), mouse anti-β-actin (1:20000, # A2228, Sigma-Aldrich), mouse anti-ICAM-1/CD54 (1:500, 1A29, #MA5407, ThermoFisher Scientific), as primary antibodies, goat anti-mouse and goat anti-rabbit (1:10000, Sigma-Aldrich) as secondary antibodies.

### Single cell force spectroscopy

Single cell force spectroscopy (SCFS) was performed with an atomic force microscope (AFM) that was connected to a ZEISS microscope Axiovert 135 M and equipped with the CellHesion^®^ 200 module (JPK Instruments, Germany). A Petri dish heater maintained the temperature at 37°C. We quantified the adhesion force (nN) and detachment energy (fJ) between single NSCLC cells and endothelial cells by means of single cell force spectroscopy. To this end we adapted our previously developed protocols [36, 37]. The CellHesion^®^ software (JPK, SPM, version 4) directed the experiment. Analysis of the data was performed using JPK Data Processing (software version 4.3.18).

Prior to all experiments, a tipless cantilever (#ARROW-TL1-50, NanoWorld AG, Switzerland) is incubated for 20 min in PBS containing 1 mg/ml wheat germ agglutinin (WGA; #L-9640, Sigma-Aldrich). WGA binds to N-acetylglucosamine on the surface of A549 cells [38] so that the cells can be “glued” to the tip of the cantilever (see below). When confluent, endothelial HMEC-1 cells (pre-incubated for 24 hours with 10 ng/ml TNF-α) are rinsed 3× with Ringer's solution of the following composition (in mmol/l: NaCl 122.5, KCl 5.4, CaCl_2_ 1.2, MgCl_2_ 0.8, HEPES 10, and D-glucose 5.5, adjusted to pH 7.4 with 1 M NaOH). Afterwards, the cells are maintained in this solution either without or with 30 μmol/l senicapoc. When indicated A549 cells are incubated with a blocking ICAM-1 antibody (10 μg/ml, # BBIG-I1, R&D systems, USA) or an IgG control antibody (8 μg/ml, # sc-2025, Santa Cruz Biotechnology, USA) on ice for 30 min prior to single cell force spectroscopy experiments. A “wound” is scratched into the cell layer using a sterile 1 ml-pipette tip. 2 μl of a A549 cell suspension is carefully pipetted to the wound area so that an individual A549 cell can be picked under optical control with the WGA-coated cantilever. To this end the cantilever is “pressed” onto a single A549 cell for 6 s (contact time) using a maximal loading force of 2 nN. The picking procedure is highly standardized to ensure that the cancer cells attached to the cantilever had always the same mechanical properties. We performed control experiments showing that the single cell force spectroscopy measurements are not confounded by large variations of cell stiffness and thus membrane tension. This is important since a soft cell at the cantilever could have a bigger contact area with an adherent endothelial cell on the bottom of the culture dish than a stiff cell. This in turn can increase the adhesion force, even if the adhesion energy per unit area is smaller [39].

Further parameters for the measurements are as follows: pulling length (z-length) was set to 100 μm (to ensure a complete separation of cells), velocity during approach and retraction was set to 5 μm/s, the spring constant of the cantilevers was 0.03 N/m. The experimental procedure is shown in Figure 1A. A cancer cell attached to the cantilever is lowered under optical control onto an endothelial cell with a maximal loading force of 1 nN. After 2 s (contact time) the cantilever is lifted and the force and energy required for detaching the A549 cell from the underlying endothelial cell can be derived from the corresponding force-distance curve (Figure 1B). Care is taken that A549 cells are lowered onto single endothelial cells of the confluent endothelial cell layer. During each experiment any given A549 cell is brought into contact with *n* = 20 individual endothelial cells. We performed at least *N* = 7 independent experiments for every condition representing ≥140 adhesion events that were analysed. For the analysis, two parameters of the force-distance curves are considered (Figure 1B): (i) the maximal adhesion-force and (ii) the area under the curve (or detachment work [fJ]). Experiments are only considered when the maximal adhesion force is ≥0.2 nN and the retraction curve has returned to the baseline by at least 50% of maximal adhesion.

### Cell-cell adhesion assay

A549 cells are incubated similar to the single cell force spectroscopy experiments with an ICAM-1 blocking antibody or an IgG control antibody on ice for 30 min. Aftherwards, 1 × 10^5^ A549 cells are added to a confluent HMEC-1 endothelial cell layer (seeded on a cover slip and placed into a 12-well plate, and pre-incubated with 10 ng/ml TNF-α for 24 hours). After 2.5 h incubation time, the cover slips are carefully washed 3× with PBS to remove all non-adherent A549 cells. Then, the cover slips are fixed with 3.5% paraformaldehyde for 20 min. Before the cover slips are covered with mounting medium (DAKO, USA), they are washed again with PBS. Images are taken using a ZEISS Observer Z microscope, linked to a camera (Andor iXon^EM^+, Visitron Systems, Germany). Quantification of adherent A549 cells is determined using ImageJ. Six independent experiments (*N* = 6) are performed in which 10 different image sections are analysed of each cover slip (*n* = 10).

### Transendothelial migration assay

To analyze the ability of the aggressive A549 cells to migrate through an endothelial cell layer, we developed a video microscopic *in vitro* “extravasation” assay (Figure 5A). In brief a thin basement membrane-like matrix (consisting 1x RPMI, 10 mmol/l HEPES, 0.04 mg/ml laminin, 0.04 mg/ml fibronectin, and 0.32 mg/ml collagen IV, adjusted to pH 7.4; [40]) is layered onto a thick collagen I layer (containing 1× RPMI, 10 mmol/l HEPES, and 0.8 mg/ml collagen I, adjusted to pH 7.4, and incubated overnight at 37°C in a 12.5 cm^2^ flask) for one hour at RT. Afterwards, 1.7 × 10^6^ HMEC-1 cells are added to the collagen double-layer and incubated for 7–10 days at 37°C, and 5% CO_2_. The medium is replaced every other day, and when the cells reach confluency, 10 ng/ml TNF-α are added. The next day, 3 × 10^5^ A549 cells and 100 ng/ml epidermal growth factor (EGF) are added either without or with senicapoc (30 μmol/l, dissolved in DMSO). Transendothelial migration is monitored over a period of 13.5 h using a ZEISS microscope Axiovert 40C, linked to a video camera (Hamamatsu, Germany). Images are taken in 5 min intervals. The analysis is performed using ImageJ. All A549 cells within the given visual field are counted in the first image of the stack (*t* = 0). Then individual cells are tracked. The criterion for transendothelial migration is that A549 cells not only settle between, but that they clearly move underneath the endothelial cells. For these cells, the time point of intrusion into the endothelium is set to represent the beginning of transendothelial migration. For the final analysis, the number of transmigrated cells is normalized to the number of A549 cells at *t* = 0. Six independent experiments were performed for each experimental condition.

### Immunofluorescence

For staining ICAM-1 in the plasma membrane of HMEC-1 cells, they are seeded on 0.5% gelatin-coated cover slips. siRNA transfection is performed as described above. For staining, the cells are washed 3x with ice-cold PBS and blocked for 1 h at 4°C with a blocking buffer containing 10 mmol/l HEPES, PBS und 1% BSA. Afterwards, the cells are incubated for 2 hours at 4°C with an antibody against ICAM-1 (# BBIG-I1, R&D systems, USA; diluted 1:250 in blocking buffer). In parallel, control slides are incubated with an anti-α-tubulin antibody (1:1000, #T6199, Sigma-Aldrich) to assess a potential permeabilization of the cells. After incubation with the primary antibody, the cells are washed twice with the blocking buffer and 2× with a PBS-buffer, containing 10 mmol/l HEPES. Then, the cells are fixed with 3.5% paraformaldehyde (PFA) in PBS for 20 min at 4°C and washed 3× with PBS. Incubation with the secondary antibody follows for 1 h at 4°C, using a goat anti-rabbit antibody labeled with Alexa Fluor 488 (1:1000, Invitrogen, Carlsbad, USA). Thereafter, the cells are washed 4× with PBS and covered with fluorescent mounting medium (DAKO, USA). Images are taken using a ZEISS Observer Z microscope, linked to a camera (Andor iXon^EM^+, Visitron Systems, Germany). Fluorescence intensity (corrected for background fluorescence) is determined using ImageJ. To ensure the membrane staining of ICAM-1 we analysed only those experiments that were negative for α-tubulin staining. Experiments are repeated three times with 10 images each.

Subsequent to the TER measurements (see below), immunofluorescence staining for VE-cadherin in HMEC-1 and HUVEC cells is performed. Cells are fixed with 2% paraformaldehyde as described above, permeabilized with 0.1% Triton X-100 in PBS (10 min at 4°C) and labeled with a primary antibody against VE-cadherin (1:100, #I1411, Santa Cruz, USA), and an Akexa488-conjugated secondary antibody (anti-goat IgG Alexa488, 1:500, #A 11055, Molecular Probes^®^ - Thermo Scientific™). Images are recorded with a ZEISS LSM780, confocal microscope system (Carl Zeiss, Göttingen, Germany).

### Transendothelial resistance measurements (TER)

Transendothelial electrical resistance (TER) of confluent endothelial cell layers was determined by means of impedance spectroscopy (ISP) as described elsewhere [35] using TER analytical hard- and software (MOS-Technologies, Telgte, Germany). Briefly, human microvascular endothelial cells (HMEC-1) or human umbilical cord vein endothelial cells (HUVEC) are grown to confluence on cross-linked gelatin in measuring chambers, and TER is automatically and continuously recorded. Measurements are performed in a standard cell culture incubator at 37°C and 5% CO_2_. TER_x_/TER_0_, the ratio between TER at a given time point *x* and TER at the time point zero, is plotted as a function of time. TER_0_ is measured just prior to the application of TNF-α.

### Biotinylation

HMEC-1 cells are grown in 10 cm dishes. After transfection with siRNA and TNF-α-treatment, dishes are placed on ice and washed 3× for 1 min with 20 ml per dish of ice-cold PBS+/+ (Sigma-Aldrich, D8662). Then PBS is replaced with 5 ml 1 mM solution of EZ-Link™ Sulfo-NHS-Biotin (Thermo Fisher Scientific, 21217) per dish and incubated for 2 h on ice. Subsequently, cells are washed 3x for 1 min with 20 ml of ice-cold Tris-buffered saline (137 mM NaCl, 2.7 mM KCl, 10 mM Tris-OH, pH 7.4) to remove unreacted biotinylation reagent. After the last wash, the fluid is replaced with 1 ml of CHAPS lysis buffer (20 mM NaCl, 140 mM KCl, 25 mM HEPES, 0.5% CHAPS (Thermo Fisher Scientific, 28300; pH 7.4). Cells are scraped off the dish, transferred to 1.5 ml reaction tube and frozen at −80°C overnight. The lysates are thawn on ice and thoroughly resuspended to ensure protein solubilization. Lysates are cleared by centrifugation at 4°C, 14000 g for 10 minutes. A 30 μl portion of each lysate is supplemented with an equal volume of 2× Laemmli buffer and frozen at −20°C as a positive loading control. 30 μl of Streptavidin bead slurry (Thermo Fisher Scientific, 20347) per lysate are washed 2× with 250 μl of mock-intracellular buffer (10 mM NaCl, 90 mM KCl, 2.2 mM MgCl_2_, 10 mM HEPES, pH 7.4) with 1 min centrifugation at 3000 g between each washing step. The supernatant is discarded so that the resin remains. The resin is resuspended and 30 μl are added to each lysate. Biotinylated surface proteins are pμlled down by incubation at 4°C with constant rocking for 60 min. Afterwards, beads are washed 3× for three minutes with 1 ml of mock-intracellular buffer for 1 min at 3000 g. The supernatant is discarded and 30 μl of the final volume of bead slurry are retained. Each sample is supplemented with 30 μl of 2× Laemmli buffer, heated for 10 minutes at 95°C and stored at −20°C.

### Statistical analysis

All experimental data are shown as mean ± SEM. Mean values of two unpaired groups were statistically analyzed by using the student's *t*-test. Mean values of more than two groups are tested using One-Way ANOVA. A *P* < 0.05 (^*^) is considered as statistically significant.

## SUPPLEMENTARY MATERIALS FIGURES


