# Supplementary material for: KCa3.1 channel inhibition leads to an ICAM-1 dependent increase of cell-cell adhesion between A549 lung cancer and HMEC-1 endothelial cells
Source: Oncotarget. 2017 Nov 28;8(68):112268–82. doi: 10.18632/oncotarget.22735 (PMC5762509; doi:10.18632/oncotarget.22735)
Supplement: Supplementary file 1 [file oncotarget-08-112268-s001.pdf]

K_{Ca}3.1 channel inhibition leads to an ICAM-1 dependent increase of cell-cell adhesion between A549 lung cancer and HMEC-1 endothelial cells

SUPPLEMENTARY MATERIALS

siRNA

KCNN4 (K_{Ca}3.1) siRNA duplex

sequence, rGrGrArGrUrUrUrArArCrArArGrGrC rArGrArGrArArGrCAC, #SR302550A, and a Universal Scrambled Negative Control, #SR30004, Origene, Rockville, USA.
